# Supplementary material for: A frequency-amplitude coordinator and its optimal energy consumption for biological oscillators
Source: Nat Commun. 2021 Oct 8;12:5894. doi: 10.1038/s41467-021-26182-2 (PMC8501100; doi:10.1038/s41467-021-26182-2)
Supplement: Supplementary file 1 — Supplementary Information [file 41467_2021_26182_MOESM1_ESM.pdf]

Supplementary Information for

A frequency-amplitude coordinator and its optimal energy consumption for biological oscillators

Bo-Wei Qin*, Lei Zhao and Wei Lin*
*** E-mail: boweiqin@fudan.edu.cn, or wlin@fudan.edu.cn**

Contents

Supplementary Note 1 Boundary conditions and corresponding solution spaces 2
Supplementary Note 2 Eigenvalues and eigenvectors of linear operator $\mathcal{L}$ 2
Supplementary Note 3 Details on Propositions 2
3.1 Proposition 1 2
3.2 Proposition2 3
Supplementary Note 4 Proofs 3
4.1 Proof of Theorem 1 3
4.2 Proof of Theorem 2 3
4.3 Proof of Theorem 3 3
4.4 Proof of Theorem 4 3
Supplementary Note 5 The principles of frequency and amplitude coordinations 3
Supplementary Note 6 Computing the normal form 4
6.1 The Neumann boundary condition 4
6.2 The Dirichlet boundary condition 6
6.3 The Robin boundary condition 6
Supplementary Note 7 Coordinations in the F-N model 7
7.1 The Neumann boundary condition 7
7.2 The Dirichlet boundary condition 8
7.3 The Robin boundary condition 9
Supplementary Note 8 Coordinations in the "cancer network" 9
Supplementary Note 9 Endogenous linear interactions in a computational model 10
Supplementary Note 10 Supplementary tables 12
Supplementary Note 11 Captions of supplementary movies 13
Supplementary Note 12 Supplementary figures 14

Supplementary Note 1 Boundary conditions and corresponding solution spaces

For Eq. (4) investigated in the main text, we consider the three boundary conditions given by

$$\begin{aligned} -\frac{\partial \mathbf{u}(0,t)}{\partial x} = \frac{\partial \mathbf{u}(\pi,t)}{\partial x} &= \mathbf{0}, \quad \text{Neumann boundary condition,} \\ \mathbf{u}(0,t) = \mathbf{u}(\pi,t) &= \mathbf{0}, \quad \text{Dirichlet boundary condition,} \\ -\frac{\partial \mathbf{u}(0,t)}{\partial x} + h\mathbf{u}(0,t) = \frac{\partial \mathbf{u}(\pi,t)}{\partial x} + h\mathbf{u}(\pi,t) &= \mathbf{0}, \quad \text{Robin boundary condition,} \end{aligned} \tag{S1}$$

where $h \in \mathbb{R}^+$. For each boundary condition, we define a real-valued Sobolev space as

$$\begin{aligned} \mathcal{H}_{\text{N.B.C.}} &= \left\{ \mathbf{u}(x) = [u_1(x), u_2(x)]^\top \;\middle|\; u_i(x) \in H^2(0,\pi),\ u_i'(0) = u_i'(\pi) = 0,\ i = 1,2 \right\}, \\ \mathcal{H}_{\text{D.B.C.}} &= \left\{ \mathbf{u}(x) = [u_1(x), u_2(x)]^\top \;\middle|\; u_i(x) \in H^2(0,\pi),\ u_i(0) = u_i(\pi) = 0,\ i = 1,2 \right\}, \\ \mathcal{H}_{\text{R.B.C.}} &= \left\{ \mathbf{u}(x) = [u_1(x), u_2(x)]^\top \;\middle|\; u_i(x) \in H^2(0,\pi),\ hu_i(0) - u_i'(0) = hu_i(\pi) + u_i'(\pi) = 0,\ i = 1,2 \right\}, \end{aligned}$$

where primes denote the differentiation with respect to x. We remark that the only difference among the three spaces is the imposed boundary condition. For the sake of convenience, hereafter, we drop the subscript of $\mathcal{H}$ and bear in mind that the space is subject to the corresponding boundary condition. Then, we also define the complexification of $\mathcal{H}$ as

$$\mathcal{H}_{\mathbb{C}} := \mathcal{H} \oplus i\mathcal{H} = \{\mathbf{u}_1 + i\mathbf{u}_2 \mid \mathbf{u}_i \in \mathcal{H},\ i = 1,2\}, \tag{S2}$$

which is equipped with the inner product

$$\langle \mathbf{p}(x), \mathbf{q}(x) \rangle = \int_0^\pi \bar{p}_1(x) q_1(x) + \bar{p}_2(x) q_2(x) \mathrm{d}x, \quad \forall\ \mathbf{p}(x),\ \mathbf{q}(x) \in \mathcal{H}_{\mathbb{C}}. \tag{S3}$$

Moreover, the norm is naturally induced by the inner product as

$$\|\mathbf{p}\| = \sqrt{\langle \mathbf{p}, \mathbf{p} \rangle},\ \forall \mathbf{p} \in \mathcal{H}_{\mathbb{C}}. \tag{S4}$$

Supplementary Note 2 Eigenvalues and eigenvectors of linear operator $\mathcal{L}$

Recall that, for each k_i described in Methods of the main text, one finds the eigenvalues λ_{k_i} of $\mathcal{L}$ by solving the eigenvalue problem

$$\mathbf{L}(k_i)\psi_{k_i} = \lambda_{k_i}\psi_{k_i}. \tag{S5}$$

Then, the eigenvector $\mathbf{q}_{k_i}(x)$ corresponding to λ_{k_i} satisfies

$$\mathcal{L}\mathbf{q}_{k_i}(x) = \lambda_{k_i}\mathbf{q}_{k_i}(x), \quad \mathbf{q}_{k_i}(x) \in \mathcal{H}_{\mathbb{C}}. \tag{S6}$$

It is written as a multiplicative of ψ_{k_i} and the Laplacian eigenfunction associated with k_i^2. Then, the expressions of $\mathbf{q}_{k_i}(x)$ for different boundary conditions are expressed in the following form

$$\begin{aligned} &\text{N.B.C.} \quad \mathbf{q}_{k_i}(x) = \psi_{k_i}\cos(k_i x), \\ &\text{D.B.C.} \quad \mathbf{q}_{k_i}(x) = \psi_{k_i}\sin(k_i x), \\ &\text{R.B.C.} \quad \mathbf{q}_{k_i}(x) = \psi_{k_i}\left[\sin(k_i x) + \frac{k_i}{h}\cos(k_i x)\right]. \end{aligned} \tag{S7}$$

We remark that the values of k_i are also different under distinct boundary conditions.

Supplementary Note 3 Details on Propositions

3.1 Proposition 1

Remember that we study the periodic oscillation arising from the Hopf bifurcation. Therefore, for the original system, there exists a critical value for the bifurcation parameter (i.e., $\epsilon = \epsilon^*$) where the Hopf bifurcation

occurs. When we make an intervention $\mathbf{F}$ on the original system, we expect that the Hopf bifurcation remains the same up to its qualitative behavior. In other words, our intervention only alter the frequency and amplitude. Therefore, there exists the same critical value for the Hopf bifurcation in the intervened system. Otherwise, the comparison between original and coordinated periodic oscillations would be unfair. Finally, the coordinated system should satisfy the condition that it possesses a critical value $\epsilon = \epsilon^*$ where the Hopf bifurcation occurs.

3.2 Proposition2

Only the most generic case (i.e., the generic Hopf bifurcation) is considered in this work, therefore, we need to avoid the complicated and degenerate case (which is not likely to occur in real applications) for the system. Consequently, at the critical bifurcation parameter, the system possesses only a pair of purely imaginary eigenvalues. Otherwise, more complicated case/bifurcation would arise. Moreover, to guarantee the stability of the investigated oscillation, the real part of every eigenvalue should not be positive. Any eigenvalue located in the right-half of the complex plane would yield an unstable periodic oscillation.

Supplementary Note 4 Proofs

4.1 Proof of Theorem 1

The Hopf bifurcation occurs when the characteristic equation has a pair of purely imaginary roots. Thus, according to Eq. (12), the critical value for the Hopf bifurcation is determined by the equation $c_1(k_i) = 0$. We then denote the non-intervened one and the coordinated one as c_1 and $\hat{c}_1$, respectively. Now, we have $\hat{c}_1 = c_1 - f_{11} - f_{22}$. It is stated in Proposition 1 that the Hopf bifurcation we consider in this work always occurs at the same bifurcation parameter, therefore, we have $c_1 = \hat{c}_1 = 0$ for all real f_{11} and f_{22}. This equation is satisfied if and only if $f_{11} + f_{12} = 0$.

4.2 Proof of Theorem 2

According to Proposition 2, there are only two cases for the roots of Eq. (12) (in the main text) for each i. The equation possesses either two negative real roots or a pair of imaginary roots whose real part is non-positive (≤ 0). For the first case, we assume that the two real solutions are: $r_1 < 0$ and $r_2 < 0$. Then, the characteristic equation has the form $\lambda_{k_i}^2 - (r_1 + r_2)\lambda_{k_i} + r_1 r_2 = 0$. Consequently, we deduce that $c_0(k_i) = r_1 r_2 > 0$. For the second case, we assume that the real part and the imaginary part are $a \leq 0$ and $b \in \mathbb{R}$, respectively. Analogously, we deduce that $c_0(k_i) = a^2 + b^2 > 0$. Finally, we conclude that $c_0(k_i)$ is strictly positive for all i.

4.3 Proof of Theorem 3

Assume that there exists $i = i^* > 0$ such that Eq. (12) possesses a pair of purely imaginary roots. Then, we deduce that $c_1(k_{i^*}) = 0$. Since $c_1(k_i)$ is strictly increasing with respect to k_i (provided that $d_1 + d_2$ is positive), we must have $c_1(k_0) < 0$. Moreover, it follows from Theorem 2 that $c_0(k_0) > 0$. Therefore, when $i = 0$, one of the roots of Eq. (12) has positive real part, and, therefore, Proposition 2 is violated. Consequently, i must be 0. This completes the proof.

4.4 Proof of Theorem 4

The Hopf bifurcation occurs when the characteristic equation possesses a pair of purely imaginary roots, which yields the equation $c_1(k_i) = 0$ [see Eq. (12) in the main text]. According to Theorem 2, we have $i = 0$ for the stable oscillation. Consequently, the bifurcation parameter for the Hopf bifurcation is determined by the equation $c_1(k_0) = 0$.

Supplementary Note 5 The principles of frequency and amplitude coordinations

As shown in the main text Fig. 3, our approach is established on the center manifold and normal form theories. A typical heterogeneity oscillation in a R-D system is depicted in the leftmost panel. The oscillation is periodic with respect to time t, therefore, for every spatial variable x, there is a parametric periodic orbit (u_1, u_2). Thus, a surface in the (x, u_1, u_2)-space is drawn. The variation along the x-axis suggests the diffusive effect caused by

the Laplacian eigenfunction (corresponding to k_0^2). Different boundary conditions have distinct eigenfunctions which lead to the surfaces with different shapes. In the second panel, the center manifold theory allows to restrict the periodic oscillation into a complex plane. In other words, the periodic oscillation is projected onto the two-dimensional eigenspace corresponding to the Hopf bifurcation. One may doubt whether it is reasonable to use a two-dimensional periodic orbit (in the complex plane) to approximate the oscillation in a three dimensional spaces [(x, u_1, u_2)-space]. Our computation (in Supplementary Note 6) shows that the variation along x-axis caused by the Laplacian eigenfunction does not have an impact on the final result of the coordination. The eigenfunction can be regarded as a coefficient and its influence is eliminated during the manipulation. Therefore, in such a way, the periodic oscillation that we need to coordinate becomes spatially homogeneous (i.e., invariant along x-axis), see the blue surface in the second panel of main text Fig. 3. Once the coordination is finally achieved, by considering the diffusive effect again, the amplitude is increased (or decreased) for all coordinates along x-axis simultaneously. Next, the normal form theory allows to obtain a circle in the complex plane which is homeomorphic to the restricted periodic orbit, see the third panel. The information of frequency and amplitude are finally collected from the normal form.

Supplementary Note 6 Computing the normal form

In this section, we introduce the detailed process [which follows the standard procedure introduced in Refs. (1) and (2)] for obtaining the normal form Eq. (9) in the main text. To do so, we first define the adjoint operator of $\mathcal{L}$ as

$$\mathcal{L}^* := \mathbf{D}\frac{\partial^2}{\partial x^2} + \mathbf{A}^\top + \mathbf{F}^\top. \tag{S8}$$

Then, we discuss the computation separately for the three boundary conditions.

6.1 The Neumann boundary condition

For the case of N.B.C., the minimal eigenvalue of the Laplacian operator is $k_0^2 = 0$. Then, the eigenvector $\mathbf{q}(x)$ is found as

$$\mathbf{q}(x) = \ell\left[1, \frac{i\omega - \xi}{a_{12} + f_{12}}\right]^\top \cos(0 \cdot x) = \ell\left[1, \frac{i\omega - \xi}{a_{12} + f_{12}}\right]^\top, \tag{S9}$$

where ℓ is a non-zero real constant which will be determined later. To apply the projection method, we take the advantage of the adjoint eigenvector $\mathbf{p}(x)$ which satisfies

$$\mathcal{L}^*\mathbf{p}(x) = \bar{\lambda}\mathbf{p}(x). \tag{S10}$$

Normalizing it by $\langle \mathbf{p}, \mathbf{q}\rangle = 1$, we obtain

$$\mathbf{p}(x) = \frac{i}{2\ell\pi\omega}\left[\xi - i\omega, a_{12} + f_{12}\right]^\top. \tag{S11}$$

We next decompose the solution $\mathbf{u}(x,t)$ as

$$\mathbf{u}(x,t) = z(t)\,\mathbf{q}(x) + \bar{z}(t)\,\bar{\mathbf{q}}(x) + \mathbf{y}(x,t), \tag{S12}$$

where $z \in \mathbb{C}$ and $\mathbf{y} \in \mathcal{H}$ satisfies $\langle \mathbf{p}, \mathbf{y}\rangle = 0,\ \forall t \in [0, +\infty)$. Then, we have

$$\begin{aligned} z &= \langle \mathbf{p}, \mathbf{u}\rangle, \\ \mathbf{y} &= \mathbf{u} - \langle \mathbf{p}, \mathbf{u}\rangle \mathbf{q} - \langle \bar{\mathbf{p}}, \mathbf{u}\rangle \bar{\mathbf{q}}, \end{aligned} \tag{S13}$$

where we have also used the fact that $\langle \mathbf{p}, \bar{\mathbf{q}}\rangle = 0$.
Before deriving the normal form of the Hopf bifurcation in $\mathbb{C}$, we first determine the value of ℓ in $\mathbf{q}(x)$. Note that the periodic solution that we investigate is restricted in the center manifold, which is tangent to the critical eigenspace spanned by $\{\Re(\mathbf{q}), \Im(\mathbf{q})\}$. If we denote the periodic solution as $\mathbf{u}_c = [u_{c,1}, u_{c,2}]^\top$, then, according to Eq. (S12), its projection onto the critical eigenspace is written as

$$\mathbf{u}_c = \mathbf{u} - \mathbf{y} = z\mathbf{q} + \bar{z}\bar{\mathbf{q}}. \tag{S14}$$

If we rewrite $z \in \mathbb{C}$ into complex and polar coordinates as $z = x_1 + ix_2 = [A\cos(\phi) + iA\sin(\phi)]/2$, with $A \geq 0$ and $\phi \in [0, 2\pi]$, then, the amplitude of x_1 and x_2 are determined by A. From Eq. (S14), we have

$$\mathbf{u}_c = [u_{c,1}, u_{c,2}]^\top = 2x_1\Re(\mathbf{q}) - 2x_2\Im(\mathbf{q}) = 2\ell\left[x_1, -\frac{\xi x_1 + \omega x_2}{a_{12} + f_{12}}\right]^\top. \tag{S15}$$

To analyze the amplitude of $u_{c,1}$, we let ℓ be 1/2. In such a way, the amplitude of $u_{c,1}$ is approximated by A. For the second element $u_{c,2}$, we have

$$u_{c,2} = -\frac{\xi x_1 + \omega x_2}{a_{12} + f_{12}} = -\frac{A}{2(a_{12} + f_{12})}[\xi \cos(\phi) + \omega \sin(\phi)] = -\frac{A}{2} \cdot \frac{\sqrt{\xi^2 + \omega^2}}{a_{12} + f_{12}} \cos(\phi + \tau), \tag{S16}$$

where $\tau = \arctan(-\omega/\xi)$. Finally, it follows from Eq. (13) (in the main text) that the amplitude of $u_{c,2}$ is approximated by

$$A\sqrt{-(a_{21} + f_{21})/(a_{12} + f_{12})}. \tag{S17}$$

Once the normal form is computed, we are able to obtain an approximation of A in terms of the system parameters and intervention intensities f_{ij}. With the same system parameters, we alter the amplitude of $u_{c,1}$ and $u_{c,2}$ by choosing different intensities. Denote by A_0 and A_c the original and coordinated amplitudes in the normal form, respectively, then, we modulate the amplitude approximately at a desired ratio given by A_c/A_0 (equivalent to r_A in the main text). However, when the modulation of A reflects back to $\mathbf{u}_c$, the amplitudes of $u_{c,1}$ and $u_{c,2}$ may not be varied at the same ratio due to the coefficient given in Eq. (S17). For instance, as mentioned in the main text, we may come across a situation that the amplitude of $u_{c,1}$ is invariant, whereas that of $u_{c,2}$ is increased or decreased. When modulating the amplitude of an ODE investigated in Ref. (3), the authors have not considered this situation and only the first state variable of the system is considered and modulated. In the present work, we want to coordinate the amplitudes of both $u_{c,1}$ and $u_{c,2}$ at the same ratio simultaneously. To this end, the intervention intensities have to satisfy

$$\sqrt{-\frac{a_{21} + f_{21}}{a_{12} + f_{12}}} = \sqrt{-\frac{a_{21} + 0}{a_{12} + 0}} = \sqrt{-\frac{a_{21}}{a_{12}}}, \tag{S18}$$

which implies Eq. (10).

We then continue computing the normal form of the Hopf bifurcation, from which the approximation of A can be obtained. Differentiating Eq. (S13) with respect to t, we have

$$\begin{aligned} \dot{z} &= \lambda z + \langle \mathbf{p}, \mathbf{g}(z\mathbf{q} + \bar{z}\bar{\mathbf{q}} + \mathbf{y}) \rangle, \\ \frac{\partial \mathbf{y}}{\partial t} &= \mathcal{L}\mathbf{y} + \frac{1}{2}\mathbf{h}_{20}z^2 + \mathbf{h}_{11}z\bar{z} + \frac{1}{2}\mathbf{h}_{02}\bar{z}^2 + \cdots, \end{aligned} \tag{S19}$$

where dots represent higher order terms. In addition, the center manifold of the Hopf bifurcation is represented as

$$\mathbf{y} = \frac{1}{2}\mathbf{w}_{20}z^2 + \mathbf{w}_{11}z\bar{z} + \frac{1}{2}\mathbf{w}_{02}\bar{z}^2 + \mathcal{O}\left(|z, \bar{z}|^3\right). \tag{S20}$$

It follows from Eq. (S19) and Eq. (S20) that

$$\begin{aligned} \frac{\partial \mathbf{y}}{\partial t} &= \mathcal{L}\left(\frac{1}{2}\mathbf{w}_{20}z^2 + \mathbf{w}_{11}z\bar{z} + \frac{1}{2}\mathbf{w}_{02}\bar{z}^2 + \cdots\right) + \frac{1}{2}\mathbf{h}_{20}z^2 + \mathbf{h}_{11}z\bar{z} + \frac{1}{2}\mathbf{h}_{02}\bar{z}^2 + \cdots \\ &= \mathbf{w}_{20}z\dot{z} + \mathbf{w}_{11}(\dot{z}\bar{z} + z\dot{\bar{z}}) + \mathbf{w}_{20}\bar{z}\dot{\bar{z}} + \cdots \\ &= \lambda\mathbf{w}_{20}z^2 + \left(\lambda + \bar{\lambda}\right)\mathbf{w}_{11}z\bar{z} + \bar{\lambda}\mathbf{w}_{20}\bar{z}^2 + \cdots. \end{aligned} \tag{S21}$$

To find the normal form, we only need to compute $\mathbf{w}_{20}$ and $\mathbf{w}_{11}$ [see Refs. (1) and (2)]. According Eq. (S21), they can be uniquely solved from

$$\begin{aligned} \mathbf{h}_{20} &= (2\lambda\mathbf{I} - \mathcal{L})\mathbf{w}_{20}, \\ \mathbf{h}_{11} &= (2\mu\mathbf{I} - \mathcal{L})\mathbf{w}_{11}, \end{aligned} \tag{S22}$$

where $\mathbf{I}$ is the 2×2 identity matrix. As discussed in Ref. (1), we expand any smooth function $\mathbf{g}(\mathbf{u})$ into the form

$$\mathbf{g}(\mathbf{u}) = \frac{1}{2}\mathbf{b}(\mathbf{u}, \mathbf{u}) + \frac{1}{6}\mathbf{c}(\mathbf{u}, \mathbf{u}, \mathbf{u}) + \cdots, \tag{S23}$$

where $\mathbf{b}(\cdot, \cdot)$ and $\mathbf{c}(\cdot, \cdot, \cdot)$ are symmetric multilinear functions. Then, $\mathbf{h}_{20}$ and $\mathbf{h}_{11}$ are computed by

$$\begin{aligned} \mathbf{h}_{20} &= \mathbf{b}(\mathbf{q}, \mathbf{q}) - \langle \mathbf{p}, \mathbf{b}(\mathbf{q}, \mathbf{q}) \rangle \mathbf{q} - \langle \bar{\mathbf{p}}, \mathbf{b}(\mathbf{q}, \mathbf{q}) \rangle \bar{\mathbf{q}}, \\ \mathbf{h}_{11} &= \mathbf{b}(\mathbf{q}, \bar{\mathbf{q}}) - \langle \mathbf{p}, \mathbf{b}(\mathbf{q}, \bar{\mathbf{q}}) \rangle \mathbf{q} - \langle \bar{\mathbf{p}}, \mathbf{b}(\mathbf{q}, \bar{\mathbf{q}}) \rangle \bar{\mathbf{q}}. \end{aligned} \tag{S24}$$

It can be easily verified that, with N.B.C., $\mathbf{h}_{20} = \mathbf{h}_{11} = \mathbf{0}$ for any smooth function $\mathbf{g}(\mathbf{u})$. Consequently, we have $\mathbf{w}_{20} = \mathbf{w}_{11} = \mathbf{0}$. Next, we expand the first equation in Eq. (S19) and rewrite it as

$$\dot{z} = \lambda z + \frac{1}{2} g_{20} z^2 + g_{11} z\bar{z} + \frac{1}{2} g_{02} \bar{z}^2 + \frac{1}{2} g_{21} z^2 \bar{z} + \mathcal{O}\left(|z|^4\right), \tag{S25}$$

where the coefficients are found as

$$g_{20} = \langle \mathbf{p}, \mathbf{b}(\mathbf{q}, \mathbf{q}) \rangle, \quad g_{11} = \langle \mathbf{p}, \mathbf{b}(\mathbf{q}, \bar{\mathbf{q}}) \rangle, \quad g_{02} = \langle \mathbf{p}, \mathbf{b}(\bar{\mathbf{q}}, \bar{\mathbf{q}}) \rangle, \tag{S26}$$

and

$$g_{21} = \langle \mathbf{p}, \mathbf{c}(\mathbf{q}, \mathbf{q}, \bar{\mathbf{q}}) \rangle + 2 \langle \mathbf{p}, \mathbf{b}(\mathbf{q}, \mathbf{w}_{11}) \rangle + \langle \mathbf{p}, \mathbf{b}(\bar{\mathbf{q}}, \mathbf{w}_{20}) \rangle = \langle \mathbf{p}, \mathbf{c}(\mathbf{q}, \mathbf{q}, \bar{\mathbf{q}}) \rangle. \tag{S27}$$

Finally, it follows from Ref. (2) that, there exists a nonlinear transformation from z to w such that Eq. (S25) can be converted into the Poincaré normal form as

$$\dot{w} = \lambda w + \eta w^2 \bar{w} + \mathcal{O}\left(|w|^4\right), \tag{S28}$$

where

$$\eta = \frac{g_{20} g_{11} \left(2\lambda + \bar{\lambda}\right)}{2|\lambda|^2} + \frac{|g_{11}|^2}{\lambda} + \frac{|g_{02}|^2}{2\left(2\lambda - \bar{\lambda}\right)} + \frac{g_{21}}{2}. \tag{S29}$$

6.2 The Dirichlet boundary condition

We now consider the D.B.C. and introduce the procedure for computing the corresponding normal form. Again, we first determine k_0. For the Laplacian operator with D.B.C., the minimal eigenvalue is $k_0^2 = 1$. Then, the ordinary and adjoint eigenvectors $\mathbf{q}(x)$ and $\mathbf{p}(x)$ are found as

$$\mathbf{q}(x) = \left[\frac{1}{2}, \frac{i\omega - \xi}{2(a_{12} + f_{12})}\right]^\top \sin(x), \;\; \mathbf{p}(x) = \frac{2i}{\pi\omega} \left[\xi - i\omega, a_{12} + f_{12}\right]^\top \sin(x), \tag{S30}$$

and they also satisfy the normalization $\langle \mathbf{p}, \mathbf{q} \rangle = 1$. Note that the eigenvectors for the D.B.C. case have the factor $\sin(x)$ so that the periodic solution arising from the Hopf bifurcation is spatially non-homogeneous and the amplitude depends on the spatial variable x. Fortunately, if one analyze the transformation of the amplitude as we do in Eqs. (S15) – (S16), it can be verified that the linear coordinator modulates the amplitude for all x at the same ratio A_c/A_0 (i.e., r_A) simultaneously. Therefore, for the same reason explained before, we set the coefficient of the first element in $\mathbf{q}(x)$ be 1/2 and the intervention intensities (f_{12} and f_{21}) also satisfy Eq. (10).

Once k_0 and the two eigenvectors $\mathbf{q}(x)$, $\mathbf{p}(x)$ are found, we can compute the normal form of the Hopf bifurcation. The procedure are exactly the same as that for the N.B.C. case, that is, computing $\mathbf{h}_{02}$ and $\mathbf{h}_{11}$ from Eq. (S24), solving $\mathbf{w}_{20}$ and $\mathbf{w}_{11}$ from Eq. (S22), calculating g_{ij} in Eq. (S25) and finally finding η in Eq. (S28).

6.3 The Robin boundary condition

Analogously, for the case of R.B.C., we determine k_0 first. By imposing the boundary condition at $x = \pi$, the minimal eigenvalue k_0^2 of the Laplacian operator satisfies

$$2k_0 h \cos(k_0\pi) + (h^2 - k_0^2)\sin(k_0\pi) = 0 \;\; \text{and} \;\; 0 < k_0 < 1. \tag{S31}$$

The ordinary eigenvector $\mathbf{q}(x)$ and normalized adjoint eigenvector $\mathbf{p}(x)$ are found as

$$\mathbf{q}(x) = \left[\frac{1}{2}, \frac{i\omega - \xi}{2(a_{12} + f_{12})}\right]^\top \cdot \left\{\sin(k_0 x) + \frac{k_0}{h}\cos(k_0 x)\right\}, \tag{S32}$$

and

$$\mathbf{p}(x) = \frac{i}{c\,\omega} \left[\xi - i\omega, a_{12} + f_{12}\right]^\top \cdot \left\{\sin(k_0 x) + \frac{k_0}{h}\cos(k_0 x)\right\}, \tag{S33}$$

where

$$c = \int_0^\pi \left[\sin(k_0 x) + \frac{k_0}{h}\cos(k_0 x)\right]^2 \mathrm{d}x = \frac{\pi}{2} + \frac{1}{h} + \frac{k_0^2 \pi}{2h^2}.$$

Note that we again let the coefficient of the first element of $\mathbf{q}(x)$ be 1/2 and take Eq. (10) into account for the same reason as before. Then, following the same procedure as described in 6.1 – 6.2, the Poincaré normal form Eq. (S28) corresponding to the R.B.C. can also be found.

We remark that, for the computation of the normal form with the three boundary conditions, the main differences are the value of k_0 and the eigenvectors $\mathbf{q}(x)$ and $\mathbf{p}(x)$. Since $\cos(0 \cdot x) = 1$ in $\mathbf{q}(x)$ for the N.B.C., the periodic oscillation arising from the Hopf bifurcation is spatially homogeneous. One of the consequences is $\mathbf{h}_{02} = \mathbf{h}_{11} = \mathbf{0}$ which makes the computation easier than those for the other two cases. For the other two cases (D.B.C. and R.B.C.), the periodic solutions are generally spatially non-homogeneous, and $\mathbf{h}_{20}$ and $\mathbf{h}_{11}$ are usually non-zero vectors. Therefore, the subsequent computations are more complicated. For all three cases, the formula of η in Eq. (S28) are extremely long. Nevertheless, to investigate a specific coordination problem in a given R-D system, the procedure introduced in this section can be easily implemented with the symbolic software, such as: *Maple*. Therefore, for the sake of simplicity, we do not show the lengthy formula here. Once the normal form coefficient η is computed, we can perform the analysis for designing the rest two intervention intensities, f_{11} and f_{12} , to design the required coordinator, which is introduced in the main text.

Supplementary Note 7 Coordinations in the F-N model

The F-N model [Eq. (14) in the main text] possesses a constant stationary solution $(V, W) = (V_0, W_0)$. Then, we find that $W_0 = V_0/\gamma$ and that V_0 is a real root of the following equation

$$\gamma V_0(V_0 - \theta)(1 - V_0) - V_0 + \gamma I = 0. \tag{S34}$$

In order to apply the procedure introduced in the main text, we introduce the following transformation

$$\tilde{V} = V - V_0, \ \tilde{W} = W - W_0, \tag{S35}$$

which moves the stationary solution to the origin and converts the original system into

$$\begin{aligned} \frac{\partial V}{\partial t} &= d_1 \frac{\partial^2 V}{\partial x^2} + \alpha V + W + \beta V^2 - V^3, \\ \frac{\partial W}{\partial t} &= \epsilon d_2 \frac{\partial^2 W}{\partial x^2} + \epsilon V - \epsilon\gamma W, \end{aligned} \tag{S36}$$

where $\alpha = -\theta + 2(\theta + 1)V_0 - 3V_0^2$ and $\beta = 1 + \theta - 3V_0$. Note that, we have dropped tildes for the sake of convenience. To achieve coordinations, by taking Theorem 1 and Eq. (10) (in the main text) into account, we introduce a coordinator comprising linear interventions as

$$\begin{bmatrix} V \to V & W \to V \\ V \to W & W \to W \end{bmatrix} := \mathbf{F} \begin{bmatrix} V \\ W \end{bmatrix} = \begin{bmatrix} f_{11} & f_{12} \\ -\epsilon f_{12} & -f_{11} \end{bmatrix} \begin{bmatrix} V \\ W \end{bmatrix}. \tag{S37}$$

Then, Eq. (S36) is in the form of Eq. (11) with

$$\mathbf{u} = [V, W]^\top, \ \mathbf{D} = \mathrm{diag}(d_1, \epsilon d_2), \ \mathbf{A} = \begin{bmatrix} \alpha & -1 \\ \epsilon & -\epsilon\gamma \end{bmatrix}, \ \mathbf{g}(\mathbf{u}) = \frac{1}{2}\mathbf{b}(\mathbf{u}, \mathbf{u}) + \frac{1}{6}\mathbf{c}(\mathbf{u}, \mathbf{u}, \mathbf{u}),$$

where

$$\mathbf{b}(\mathbf{u}, \mathbf{u}) := [2\beta V^2, 0]^\top, \ \mathbf{c}(\mathbf{u}, \mathbf{u}, \mathbf{u}) = [-6V^3, 0]^\top.$$

Next, we follow the analysis performed in sections 6.1 – 6.3 (in Supplementary Information) to design the matrix $\mathbf{F}$ and to coordinate frequency and amplitude for different type of boundary conditions.

7.1 The Neumann boundary condition

We first investigate the system with N.B.C. given in Eq. (S1). It follows from Theorem 4 (in the main text) and the analysis in 6.1 (in Supplementary Information) that the critical value, at which the required Hopf bifurcation occurs, is solved from

$$c_1(k_0) = c_1(0) = \epsilon\gamma - \alpha. \tag{S38}$$

Here, we regard ϵ as the bifurcation parameter. Accordingly, the Hopf bifurcation occurs at $\epsilon^* = \alpha/\gamma$. Then, when ϵ is sufficiently close to ϵ^*, the unique pair of pure imaginary eigenvalues of the linear operator $\mathcal{L}$ is written as

$$\lambda = \mu + i\omega, \ \bar{\lambda} = \mu - i\omega \text{ with } \omega > 0, \tag{S39}$$

where

$$\mu = \frac{1}{2}(\alpha - \epsilon\gamma), \quad \omega^2 = \epsilon(1-f_{12})^2 - \xi^2, \tag{S40}$$

with $\xi = f_{11} + (\alpha + \epsilon\gamma)/2$. The ordinary eigenvector $\mathbf{q}(x)$ and the adjoint eigenvector $\mathbf{p}(x)$ are found as

$$\mathbf{q}(x) = \left[\frac{1}{2}, \frac{\xi - i\omega}{2(1-f_{12})}\right]^\top, \quad \mathbf{p}(x) = \frac{i}{\pi\omega}\left[\xi - i\omega, f_{12} - 1\right]^\top. \tag{S41}$$

Then, applying the formulas given 6.1, we obtain

$$g_{02} = g_{11} = g_{20} = \frac{\beta}{2}\left(1 - i\frac{\xi}{\omega}\right), \quad g_{21} = -\frac{3}{4}\left(1 - i\frac{\xi}{\omega}\right), \tag{S42}$$

and the real part of the normal form coefficient is calculated as

$$\chi(f_{11}, f_{12}) = \Re(\eta) = -\frac{3}{8} + \frac{\beta^2}{4(\mu^2+\omega^2)}\left[f_{11} + 2\alpha - \epsilon\gamma - 4\mu\frac{\epsilon(1-f_{12})^2}{\mu^2 + 9\omega^2}\right]. \tag{S43}$$

Then, to achieve independent frequency coordination and to obtain required frequency, we set $r_A = 1$ and a free r_F. For a desired r_F, we solve the two algebraic equations (mentioned in main text section "A universal design policy for the coordinator") to choose the appropriate values of f_{11} and f_{12}. They can be easily solved numerically. To achieve independent amplitude coordination, we set $r_F = 1$ and a free r_A.

7.2 The Dirichlet boundary condition

If the D.B.C. given in Eq. (S1) is imposed, we then have $k_0 = 1$, and the critical value of the bifurcation parameter is solved from $c_1(1) = 0$ as

$$\epsilon^* = \frac{\alpha - d_1}{\gamma + d_2}. \tag{S44}$$

When ϵ is sufficiently close to ϵ^*, the two eigenvalues become

$$\lambda = \mu + i\omega, \ \bar{\lambda} = \mu - i\omega \text{ with } \omega > 0, \tag{S45}$$

where

$$\mu = \frac{1}{2}\left[\alpha - d_1 - \epsilon(\gamma + d_2)\right], \quad \omega^2 = \epsilon(1-f_{12})^2 - \xi^2, \tag{S46}$$

with $\xi = f_{11} + \frac{1}{2}\left[\alpha - d_1 + \epsilon(\gamma + d_2)\right]$. The corresponding ordinary and adjoint eigenvectors are found as

$$\mathbf{q}(x) = \left[\frac{1}{2}, \frac{\xi - i\omega}{2(1-f_{12})}\right]^\top \sin(x), \ \mathbf{p}(x) = \frac{2i}{\pi\omega}\left[\xi - i\omega, f_{12} - 1\right]^\top \sin(x). \tag{S47}$$

We then follow the procedure introduced in Supplementary Note 6 and obtain

$$\mathbf{h}_{20} = \mathbf{h}_{11} = \frac{\beta}{4}\left[1 - \frac{16}{3\pi}\sin(x) - \cos(2x)\right] \cdot \begin{bmatrix}1\\0\end{bmatrix}. \tag{S48}$$

As mentioned at the end of Supplementary Note 6 (in Supplementary Information), $\mathbf{h}_{20}$ and $\mathbf{h}_{11}$ are non-zero vectors. We note that they can be decomposed as $\mathbf{h}^{(0)} + \mathbf{h}^{(1)}\sin(x) + \mathbf{h}^{(2)}\cos(2x)$. Thus, $\mathbf{w}_{20}$ and $\mathbf{w}_{11}$ solved from Eq. (S22) can be written as

$$\begin{aligned}\mathbf{w}_{20} &= \mathbf{w}_{20}^{(0)} + \mathbf{w}_{20}^{(1)}\sin(x) + \mathbf{w}_{20}^{(2)}\cos(2x),\\ \mathbf{w}_{11} &= \mathbf{w}_{11}^{(0)} + \mathbf{w}_{11}^{(1)}\sin(x) + \mathbf{w}_{11}^{(2)}\cos(2x),\end{aligned} \tag{S49}$$

where the vector coefficients are obtained by computing

$$\mathbf{w}_{20}^{(j)} = [2\lambda - \mathbf{L}(j^2)]^{-1}\mathbf{h}^{(j)} \quad \text{and} \quad \mathbf{w}_{11}^{(j)} = [2\mu - \mathbf{L}(j^2)]^{-1}\mathbf{h}^{(j)}, \ \ j = 0, 1, 2. \tag{S50}$$

Once $\mathbf{w}_{20}$ and $\mathbf{w}_{11}$ are found, g_{ij} are computed from Eqs. (S26) – (S27), and then the normal form coefficient η is calculated by Eq. (S29). Although the expression of η is too long to be written here, it can be easily manipulated by symbolic softwares. For instance, we use *Maple* to implement the computation. Every computed formula is always stored in the memory and can be called whenever it is needed. Therefore, it is not necessary to know the exact expression of η during the investigations.

7.3 The Robin boundary condition

Finally, we consider the R.B.C. given in Eq. (S1). In this case, k_0 is solved from Eq. (S31) and the critical value for the Hopf bifurcation is obtained as

$$\epsilon^* = \frac{\alpha - d_1 k_0^2}{\gamma + d_2 k_0^2}. \tag{S51}$$

For ϵ is sufficiently close to ϵ^*, the pair of purely imaginary eigenvalues of $\mathcal{L}$ is written as

$$\lambda = \mu + i\omega, \ \bar{\lambda} = \mu - i\omega \text{ with } \omega > 0, \tag{S52}$$

where

$$\mu(\epsilon) = \frac{1}{2}\left[\alpha - d_1 k_0^2 - \epsilon(\gamma + d_2 k_0^2)\right], \quad \omega^2(\epsilon) = \epsilon(1 - f_{12})^2 - \xi^2,$$

with $\xi = f_{11} + \frac{1}{2}\left[\alpha - d_1 k_0^2 + \epsilon(\gamma + d_2 k_0^2)\right]$. The corresponding ordinary and adjoint eigenvectors follow Eq. (S32) and Eq. (S33), respectively. From Eq. (S24), we then obtain

$$\mathbf{h}_{20} = \mathbf{h}_{11} = \frac{\beta}{4h^2}\left\{ \begin{bmatrix} h^2 + k_0^2 \\ 0 \end{bmatrix} + s \begin{bmatrix} 1 \\ 0 \end{bmatrix} \left[\sin(k_0 x) + \frac{k_0}{h}\cos(k_0 x)\right] + 2\begin{bmatrix} k_0 h \\ 0 \end{bmatrix} \sin(2k_0 x) + \begin{bmatrix} k_0^2 - h^2 \\ 0 \end{bmatrix} \cos(2k_0 x) \right\}, \tag{S53}$$

where

$$s = \frac{2[\cos(k_0\pi)(k_0^2 + h^2) + (k_0^2 - h^2)]}{3ck(k_0^2 - h^2)}[\cos^2(k_0\pi)(k_0^2 + h^2) - \cos(k_0\pi)(k_0^2 - h^2) - 3k_0^2 - 2h^2].$$

Analogous to the D.B.C. case, $\mathbf{h}_{20}$ and $\mathbf{h}_{11}$ are regarded as a combination of several components, namely, $\cos(j \cdot k_0 x)$ and $\sin(j \cdot k_0 x)$ with $j = 0, 1, 2$. Then, we find $\mathbf{w}_{20}$ and $\mathbf{w}_{11}$ in the same manner and need to compute

$$[2\lambda - \mathbf{L}(j^2 \cdot k_0^2)]^{-1} \quad \text{and} \quad [2\mu - \mathbf{L}(j^2 \cdot k_0^2)]^{-1}, \ \ j = 0, 1, 2, \tag{S54}$$

for the corresponding component. Having obtained $\mathbf{w}_{20}$ and $\mathbf{w}_{11}$, we then compute the normal form coefficient η by applying the same formulas as before.

Once the Poincaré normal form is obtained, the coordinations of frequency and amplitude are straightforward following the procedure introduced in the main text.

Supplementary Note 8 Coordinations in the "cancer network"

The spatially homogeneous version (ODE) of the "cancer network" model considered in the main text [Eq. (1)] is formulated by Aguda et al. (4). The model consists of two components, namely, a protein module (Myc and E2Fs) and a micro-RNA cluster (miR-17-92). The miR-17-92 is either oncogenic or tumor suppressive, and the levels of protein alters mammalian cell cycle and further affect the probability of oncogenesis. Therefore, the model can be used to explain the mechanisms of micro-RNA in a "cancer network". The steady-state of nondimensionalized model was well analyzed by the authors, and it was shown that the levels of protein module may enter a cancer zone with chosen system parameters. By further considering molecular diffusion, in (5), Zheng et al. investigated a R-D version of the model. Here, we consider oscillations in the nondimensionalized system including diffusion terms and try to control their frequencies or amplitudes via the Michaelis-Menten regulations. In Eq. (1), all system parameters are positive. Their definition can be found in Ref. (4).

To coordinate the frequency and amplitude of the periodic solution arising from the quiescent state, we take ϵ as the bifurcation parameter and introduce the Michaelis-Menten regulations [Eq. (2) in the main text]. To determine the coefficients f_{ij}, we consider a linear approximation as

$$\begin{bmatrix} \phi \to \phi & \mu \to \phi \\ \phi \to \mu & \mu \to \mu \end{bmatrix} := \mathbf{F}\begin{bmatrix} \phi - \phi_0 \\ \mu - \mu_0 \end{bmatrix} = \begin{bmatrix} f_{11} & f_{12} \\ f_{21} & -f_{11} \end{bmatrix}\begin{bmatrix} \phi - \phi_0 \\ \mu - \mu_0 \end{bmatrix}, \tag{S55}$$

where we have applied Theorem 1. Note that the coefficients f_{ij} in the above matrix and those in Eq. (2) differ by Michaelis constants K_{ij}. By introducing the transformation $[\tilde{\phi}, \tilde{\mu}]^\top = [\phi - \phi_0, \mu - \mu_0]^\top$ and applying the Taylor expansion about $(\tilde{\phi}, \tilde{\mu}) = (0, 0)$, we then translate the system into the form of Eq. (11) with (for the sake

of convenience, we drop the tildes)

$$\mathbf{u}=[\phi,\mu]^\top,\ \mathbf{D}=\mathrm{diag}(d_p,d_m),\ \mathbf{A}=\begin{bmatrix}a_{11} & a_{12}\\ a_{21} & a_{22}\end{bmatrix},\ \mathbf{g}(\mathbf{u})=\frac{1}{2}\mathbf{b}(\mathbf{u},\mathbf{u})+\frac{1}{6}\mathbf{c}(\mathbf{u},\mathbf{u},\mathbf{u})+\cdots,$$

$$\mathbf{b}(\mathbf{u},\mathbf{u})=\frac{2\kappa}{\epsilon(\Gamma_1'+\phi_0^2+\Gamma_2'\mu_0)^3}[B_{20}\phi^2+B_{11}\phi\mu+B_{02}\mu^2,0]^\top,$$

$$\mathbf{c}(\mathbf{u},\mathbf{u},\mathbf{u})=\frac{6\kappa}{(\Gamma_1'+\phi_0^2+\Gamma_2'\mu_0)^4}[C_{30}\phi^3+C_{21}\phi^2\mu+C_{12}\phi\mu^2+C_{03}\mu^3,0]^\top,$$

where

$$a_{11}=-\frac{1}{\epsilon}+2\frac{\kappa\phi_0(\Gamma_1'+\Gamma_2'\mu_0)}{\epsilon(\Gamma_1'+\phi_0^2+\Gamma_2'\mu_0)^2},\ a_{12}=-\frac{\kappa\Gamma_2'\phi_0^2}{\epsilon(\Gamma_1'+\phi_0^2+\Gamma_2'\mu_0)^2}, a_{21}=1,\ a_{22}=-1,$$

$$B_{20}=(\Gamma_1'-3\phi_0^2+\Gamma_2'\mu_0)(\Gamma_1'+\Gamma_2'\mu_0),\ B_{11}=-2\Gamma_2'\phi_0(\Gamma_1'-\phi_0^2+\Gamma_2'\mu_0),\ B_{02}=(\Gamma_2')^2\phi_0^2,$$

$$C_{30}=-4\phi_0(\Gamma_1'+\Gamma_2'\mu_0)(\Gamma_1'-\phi_0^2+\Gamma_2'\mu_0),\ C_{21}=\Gamma_2'[\phi_0^2(8\Gamma_1'-3\phi_0^2+8\Gamma_2'\mu_0)-(\Gamma_1'+\Gamma_2'\mu_0)^2],$$

$$C_{12}=2(\Gamma_2')^3\phi_0(\Gamma_1'-2\phi_0^2+\Gamma_2'\mu_0),\ C_{03}=-(\Gamma_1')^3\phi_0^2.$$

As an example, in the present work, we impose the R.B.C. with $h=1$ to investigate the periodic oscillation. We then find $k_0\approx 0.6383$ from Eq. (S31). Following Theorem 4 (in the main text), we obtain the critical value for the required Hopf bifurcation as

$$\epsilon^*=\frac{1}{k_0^2(d_p+d_m)+1}\left[\frac{2\kappa\phi_0(\Gamma_1'+\Gamma_2'\mu_0)}{(\Gamma_1'+\phi_0^2+\Gamma_2'\mu_0)^2}-1\right]. \tag{S56}$$

According to Eq. (10), the intervention intensity f_{21} is designed as

$$f_{21}=\frac{a_{21}}{a_{12}}f_{12}=-\frac{\epsilon(\Gamma_1'+\phi_0^2+\Gamma_2'\mu_0)^2}{\kappa\Gamma_2'\phi_0^2}f_{12}. \tag{S57}$$

Again, we then follow the procedure given in Supplementary Note 6 (in Supplementary Information) and implement the computation in *Maple* to obtain the coefficients in the Poincaré normal form and to perform required coordinations.

Supplementary Note 9 Endogenous linear interactions in a computational model

For a given biochemical oscillator, the endogenous linear interactions always exist, and they are extractable from the corresponding computational model. Moreover, the intensities of these linear interactions are determined (or interfered) by the system parameters, such as: the intensity of an upstream-regulating signal, synthesis rate, degradation rate, etc. Here, we illustrate the kinetic model of an RNA-protein negative-feedback loop as an example [see Ref. (6)]. Its reaction-diffusion version is written as:

$$\begin{aligned}\frac{\partial X(x,t)}{\partial t}&=d_x\frac{\partial^2 X(x,t)}{\partial x^2}+k_1S\frac{K_d^p}{K_d^p+Y^p}-k_{dx}X,\\ \frac{\partial Y(x,t)}{\partial t}&=d_y\frac{\partial^2 Y(x,t)}{\partial x^2}+k_{sy}X-k_{dy}Y-k_2E_T\frac{Y}{K_m+Y+K_IY^2}.\end{aligned} \tag{S58}$$

In this model, X and Y represent the expression level of mRNA and protein, respectively. There are 13 parameters involved in this system whose physical meanings are listed in Table 1 .

Table 1: The parameters and their physical meanings.

Parameter	Physical meaning	Parameter	Physical meaning
S	intensity of an upstream-regulating signal	k_1	synthesis rate of mRNA
p	integer indicating monomer, dimer, trimer, etc.	k_d	dissociation constant
k_{dx}	degradation rate of mRNA	k_{sy}	synthesis rate of protein
k_{dy}	(linear) degradation rate of protein	k_2	enzymatic degradation rate of protein
E_T	concentration of enzyme	K_m	Michaelis constant
K_I	inhibition constant of enzymatic degradation	d_x	diffusion constant of mRNA
d_y	diffusion constant of protein		

This generic model describes a simple oscillator involving one mRNA and its corresponding protein. The Hopf bifurcation and a periodic oscillation exist under certain parameter values. As mentioned in the main text, the linear interaction matrix **A** is the Jacobian matrix at the equilibrium $(X,Y) = (X^*,Y^*)$, where $X^* = k_1 S K_d^p/[k_{dx}(K_d^p + Y^{*p})]$ and Y^* is the real root of the following equation

$$-k_{dy}Y^* + \frac{k_{sy}k_1 S k_d^p}{(k_d^p + Y^{*p})k_{dx}} + \frac{k_2 E_T Y^*}{K_m + Y^* + K_I {Y^*}^2} = 0. \tag{S59}$$

Then, by simple manipulation, the linear interaction matrix is found as

$$\mathbf{A} = \begin{bmatrix} a_{11} & a_{12} \\ a_{21} & a_{22} \end{bmatrix} = \begin{bmatrix} -k_{dx} & -\frac{k_1 S k_d^p p}{(k_d^p+Y^{*p})^2} Y^{*p-1} \\ k_{sy} & -k_{dy} - \frac{k_2 E_T}{K_m+Y^*+K_I {Y^*}^2} + \frac{k_2 E_T Y^*(1+2K_I Y^*)}{(K_m+Y^*+K_I {Y^*}^2)^2} \end{bmatrix}. \tag{S60}$$

Accordingly, the endogenous linear interactions are indeed determined by the system parameters. The relation between the linear interactions a_{ij} and system parameters are given in Table 2. If a small perturbation is added on system parameter, then the intensity of the associated linear interaction is also changed. For instance, a small variation made on the concentration of enzyme E_T yields the change of a_{22}. Such a variation can be regarded as an intervention which further affects the frequency and amplitude of the oscillation arising from the Hopf bifurcation. In Fig. 1, the diagram of the linear interactions at the Hopf bifurcation is depicted. The parameters are chosen following the values provided in Ref. (6).

Table 2: The linear interactions and their related system parameters.

Linear interaction	System parameters
a_{11}	k_{dx}
a_{12}	$k_1,\ S,\ k_d,\ p,\ Y^*$
a_{21}	k_{sy}
a_{22}	$k_{dy},\ k_2,\ E_T,\ K_m,\ K_I,\ Y^*$

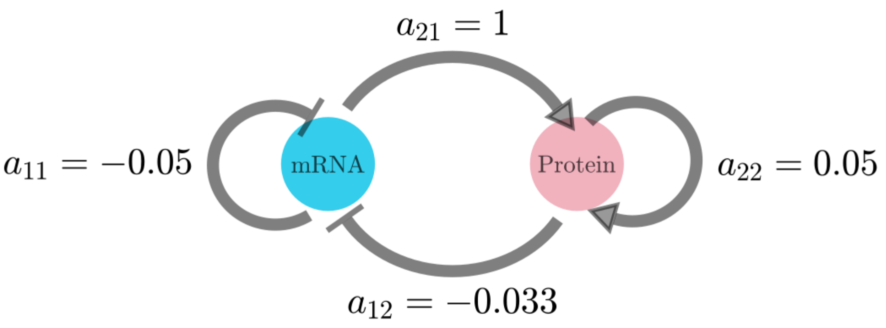

Figure 1: The linear interactions a_{ij} in the computational model Eq. (S58). The parameters are set as: $k_1 = k_{dx} = k_{dy} = 0.05$, $p = 4$, $K_m = 0.1$, $k_d = 1$, $K_I = 2$, $k_{sy} = 1$, $k_2 = 1$ and $E_T = 1$. Positive and negative interactions are indicated by arrows and I-shaped curves, respectively.

Supplementary Note 10 Supplementary tables

Table 3: The parameters used in the main text and the information of the periodic oscillation for the "cancer network".

**Parameter**	**Value**
Γ_1'	1
Γ_2'	2.5
κ	5
α'	0.35
d_p	1
d_m	1
Equilibrium (ϕ_0, μ_0)	$(7/4, 11/4)$
Hopf bifurcation ϵ^*	0.08375
The chosen (small) oscillation at ϵ	0.08
The chosen (big) oscillation at ϵ	0.05
Boundary condition	R.B.C with $h = 1$
f_{21} in the coordinator	$-25\epsilon f_{12}/8$
f_{22} in the coordinator	$-f_{11}$

Table 4: The intensities f_{ij} used in Fig. 2 of the main text.

**Figure # and time label t**	**Intensities $(f_{11}, f_{12}, f_{21}, f_{22})$ used in Eq. (2) of the main text**
Fig. 2a-d, $t = 50$	$(1.2718, -2.9073, 0.7268, -1.2718)$
Fig. 2a-d, $t = 100$	$(3.0199, -6.8024, 1.7006, -3.0199)$
Fig. 2a-d, $t = 150$	$(5.5236, -12.2476, 3.0619, -5.5236)$
Fig. 2a-d, $t = 200$	$(9.3067, -20.2937, 5.0734, -9.3067)$
Fig. 2a-d, $t = 250$	$(15.4536, -33.1114, 8.2778, -15.4536)$
Fig. 2e-f, $t = 50$	$(2.5000, -5.0512, 0.7892, -2.5000)$
Fig. 2e-f, $t = 100$	$(5.0000, -10.5335, 1.6459, -5.0000)$
Fig. 2e-f, $t = 150$	$(7.5000, -16.2245, 2.5351, -7.5000)$
Fig. 2e-f, $t = 200$	$(10.0000, -22.0583, 3.4466, -10.0000)$
Fig. 2e-f, $t = 250$	$(12.5000, -27.9931, 4.3739, -12.5000)$

Table 5: The parameters used in the main text and the information of the periodic oscillation for the F-N system.

**Boundary condition** / **Parameter**	Neumann	Dirichlet	Robin ($h = 1$)
d_1	1	0.01	0.5
d_2	1	0.01	0.5
θ	0.2	0.2	0.2
γ	2.5	2.5	2.5
I	0.1	0.1	0.1
Equilibrium (V_0, W_0)	$(0.3068, 0.1227)$	$(0.3068, 0.1227)$	$(0.3068, 0.1227)$
Hopf bifurcation ϵ^*	0.10157	0.09718	0.018563
The chosen oscillation at ϵ	0.1	0.096	0.018 and 0.01

Supplementary Note 11 Captions of supplementary movies

Table 6: Captions of supplementary movies.

Number	Caption
Movie 1	This animation shows the independent frequency coordination of the periodic oscillation in the F-N system with zero flux at the boundaries. The two coefficients for the applied coordinator are shown in the top two panels. In the bottom, we see that, as the coordination varies (the moving circle), the frequency is decreased while the amplitude is almost unchanged.
Movie 2	This animation shows the independent frequency coordination of the periodic oscillation in the F-N system with fixed value at the boundaries. The two coefficients for the applied coordinator are shown in the top two panels. In the bottom, we see that, as the coordination varies (the moving circle), the frequency is decreased while the amplitude is almost unchanged.
Movie 3	This animation shows the independent frequency coordination of the periodic oscillation in the F-N system with the Robin boundary condition. It can be regarded as the intermediate case between the case shown in Movie 1 and 2. For this case, the two coefficients for the coordinator are shown in the top two panels. In the bottom, we see that, as the coordination varies (the moving circle), the frequency is decreased while the amplitude is almost unchanged.
Movie 4	This animation shows the independent amplitude coordination of the periodic oscillation in the F-N system with zero flux at the boundary (N.B.C.). As the coordinator varies (the moving circle), the amplitude is suppressed (the color becomes darker) while the frequency is almost unchanged. For convenience, we also show the time course at $x^* = \pi/2$ in the bottom.
Movie 5	This animation shows the independent amplitude coordination of the periodic oscillation in the "cancer network". With different coordinating policies (the moving circle) shown in the top panels, the amplitude of the protein concentration is suppressed (bottom panel). Note that A_0/A_c equals the reciprocal of r_A.
Movie 6	This animation shows the phase space evolution of the time course in Fig. 7b. Six panels show the evolution in different time intervals. Obviously, due to the hybrid coordination, the amplitude (size of the circle) and the frequency are different in these panels.

Supplementary Note 12 Supplementary figures

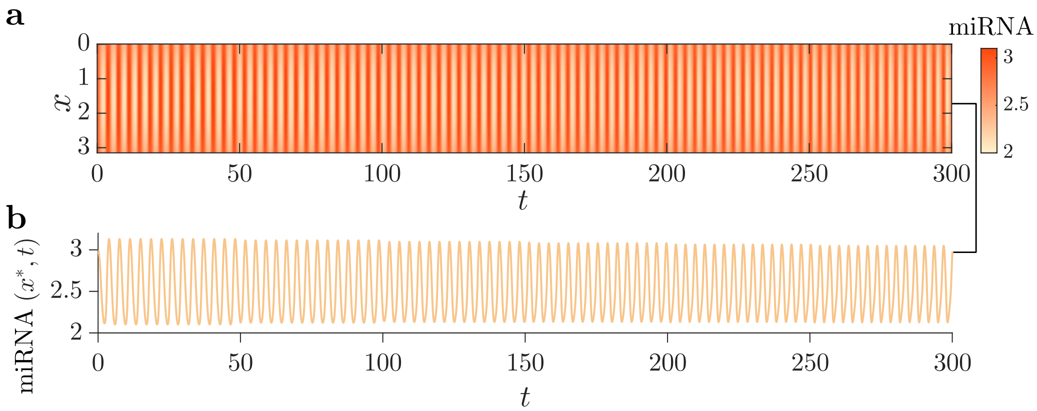

Figure 2: **a** The time course of miRNA cluster for independent amplitude coordination of the "bigger" oscillation in the "cancer network", which corresponds to the time course of protein concentration given in the main text Fig. 2e, f. **b** The time course at $x^* = \pi/2$.

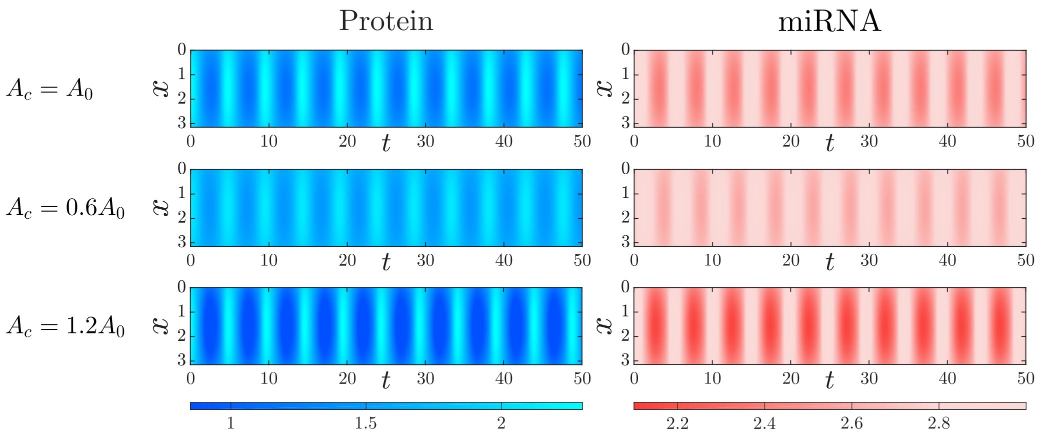

Figure 3: Independent amplitude coordinations for the periodic oscillation (close to the quiescent state) in the "cancer network". The first row shows the original oscillation without a coordinator. In the second row, the amplitude is suppressed ($r_A = 0.6$) by the MM regulations $[(f_{11}, f_{12}) = (-8.133, 6.513)]$. In the last row, the amplitude is magnified ($r_A = 1.2$) by the MM regulations $[(f_{11}, f_{12}) = (10.465, -18.764)]$.

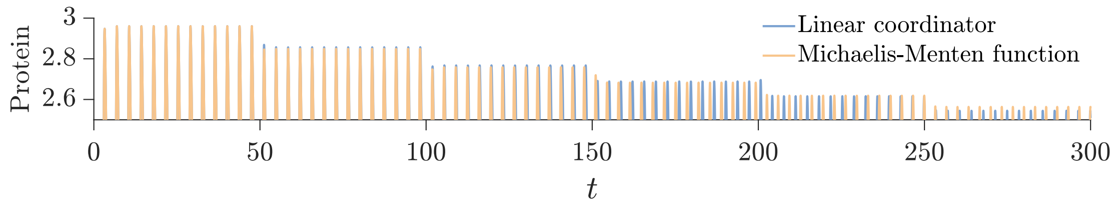

Figure 4: Reduce the amplitude of the "bigger" oscillation in the "cancer network" using distinct coordinators. The orange curve is the same as that shown in the main text Fig. 2f representing the time course coordinated by the MM functions, while the blue one is coordinated using linear terms alone. Apparently, the two coordinators have almost the same effect on coordinating the amplitude.

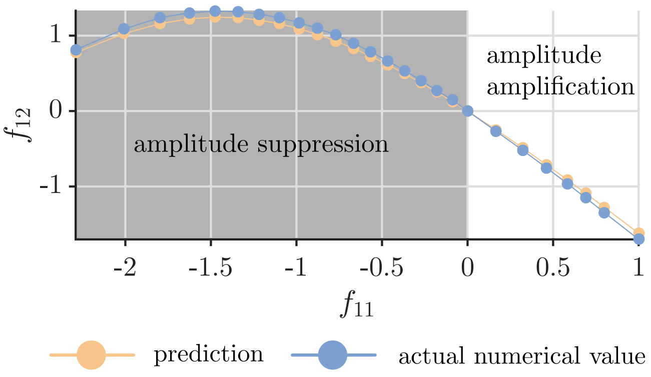

Figure 5: We compared the coordinator estimated by our computational approach with the one found numerically (in the linear regime). The coordinator was applied to the oscillation (close to the quiescent state) in the "cancer network". The theoretical prediction and numerical verification are depicted in orange and blue, respectively.

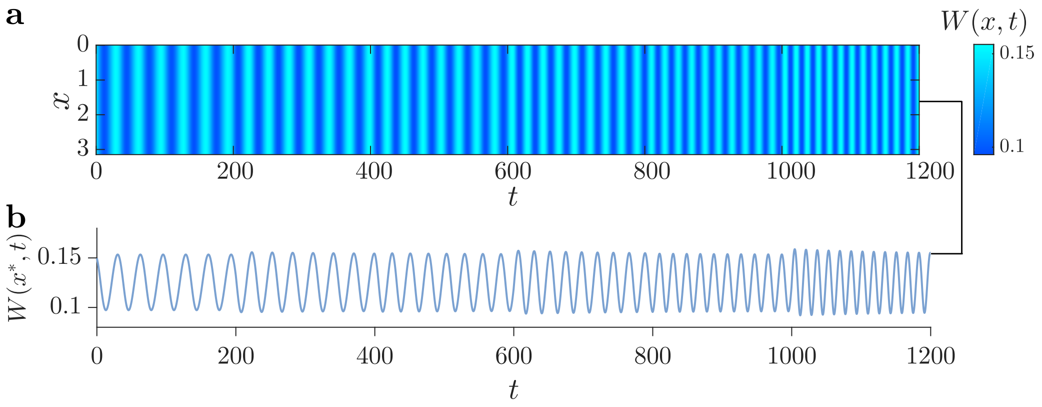

Figure 6: **a** The time course of component W for independent frequency coordination in the F-N system with N.B.C., which corresponds to the time course of V given in the main text Fig. 4a. **b** The time course at $x^* = \pi/2$.

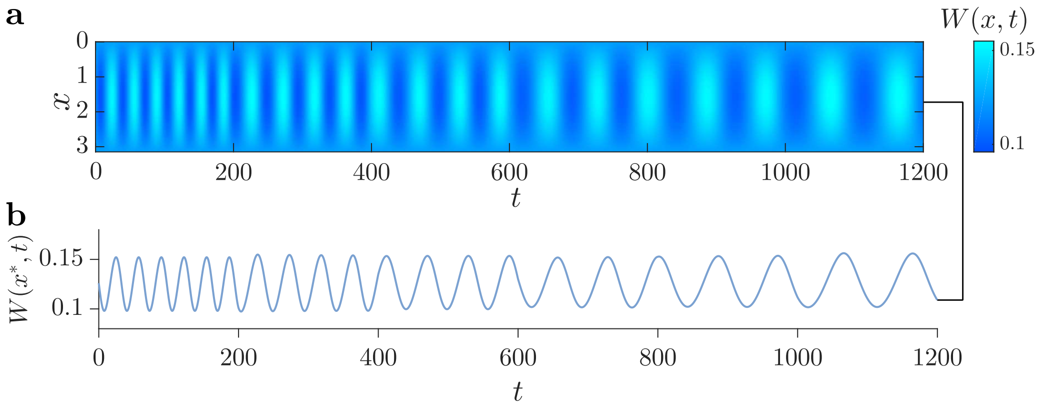

Figure 7: **a** The time course of component W for independent frequency coordination in the F-N system with D.B.C., which corresponds to the time course of V given in the main text Fig. 4f, g. **b** The time course at $x^* = \pi/2$.

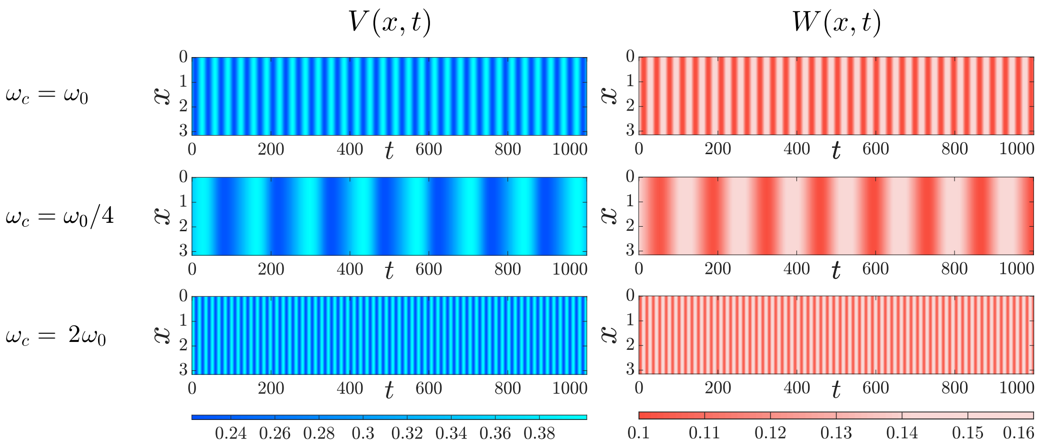

Figure 8: Independent frequency coordination in the F-N system with N.B.C. The first row shows the original oscillation without a coordinator. In the second row, the frequency is quartered by the coordinator $(f_{11}, f_{12}) = (-0.2409, 1.1550)$. In the last row, the frequency is doubled by the coordinator $(f_{11}, f_{12}) = (0.7710, 4.4532)$.

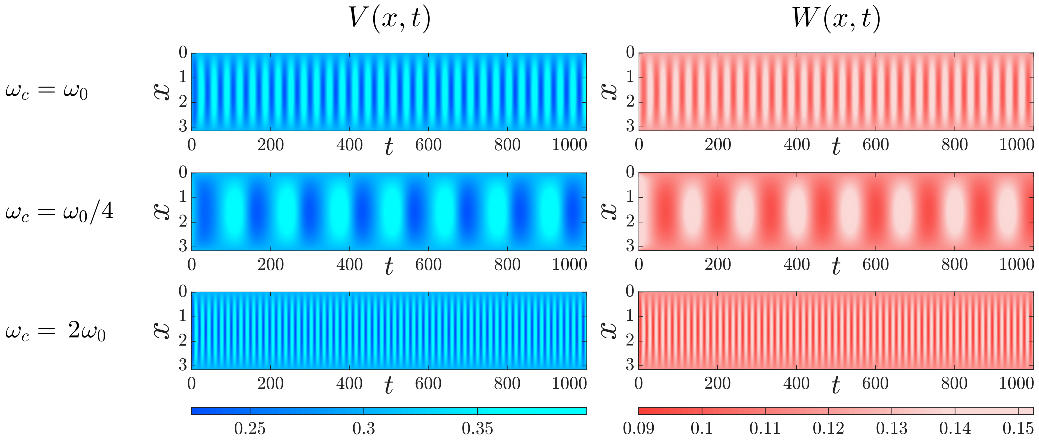

Figure 9: Independent frequency coordination in the F-N system with D.B.C. The first row shows the original oscillation without a coordinator. In the second row, the frequency is quartered by the coordinator $(f_{11}, f_{12}) = (-0.2327, 0.8412)$. In the last row, the frequency is doubled by the coordinator $(f_{11}, f_{12}) = (0.7504, -2.4379)$.

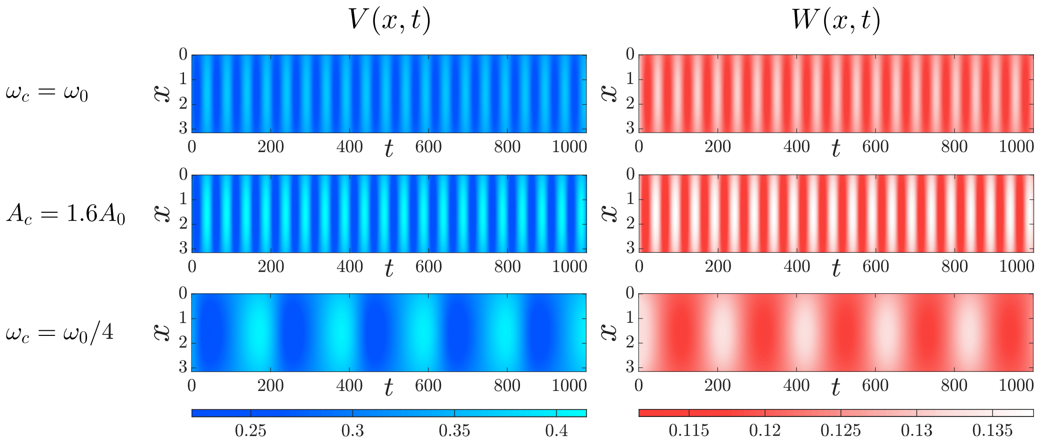

Figure 10: Independent amplitude and frequency coordinations for the periodic oscillation (at $\epsilon = 0.018$) in the F-N system with R.B.C. The first row shows the original oscillation without a coordinator. In the second row, the amplitude is magnified ($r_A = 1.6$) by the coordinator $(f_{11}, f_{12}) = (0.2, -1.0872)$. In the last row, the frequency is quartered by the coordinator $(f_{11}, f_{12}) = (-0.047, 0.7669)$.

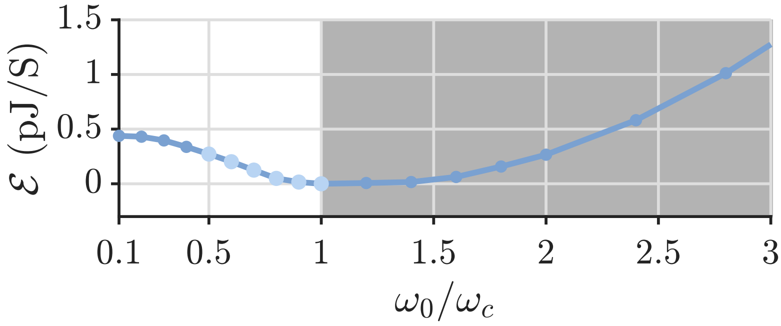

Figure 11: The average energy consumption of the optimal coordinator for independent frequency coordination. It is a zoom-in view of the blue curve shown in Fig. 4e in the main text.

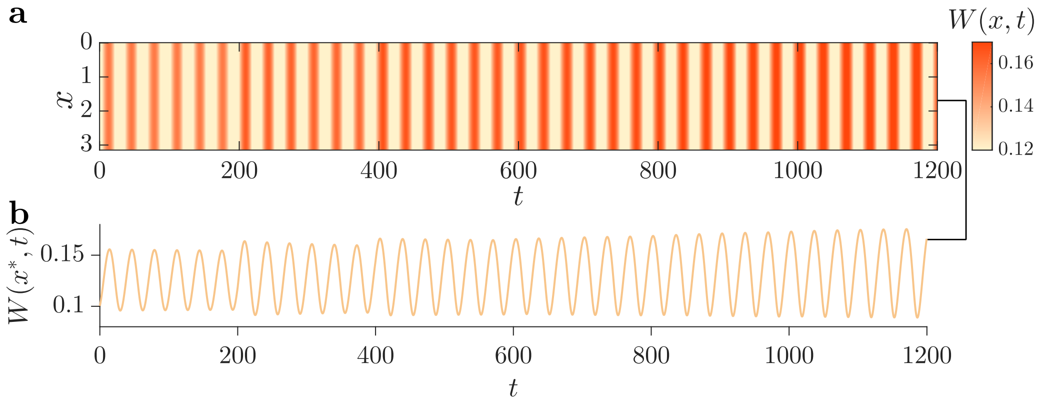

Figure 12: **a** The time course of component W for independent amplitude coordination in the F-N system with N.B.C., which corresponds to the time course of V given in main text Fig. 5a. **b** The time course at $x^* = \pi/2$.

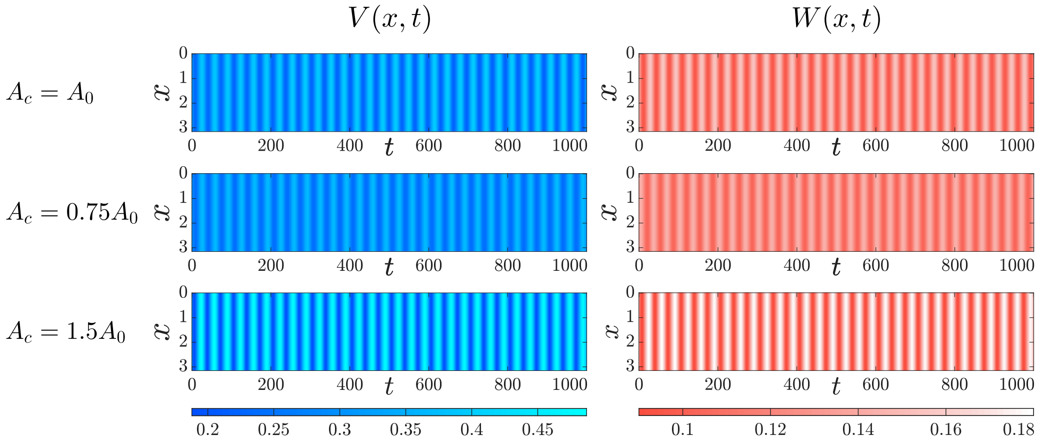

Figure 13: Independent amplitude coordinations for the periodic oscillation in the F-N system with N.B.C. The first row shows the original oscillation without a coordinator. In the second row, the amplitude is suppressed ($r_A = 0.75$) by the coordinator $(f_{11}, f_{12}) = (-0.4, 0.2356)$. In the last row, the amplitude is magnified ($r_A = 1.5$) by the coordinator $(f_{11}, f_{12}) = (0.45, -1.3006)$.

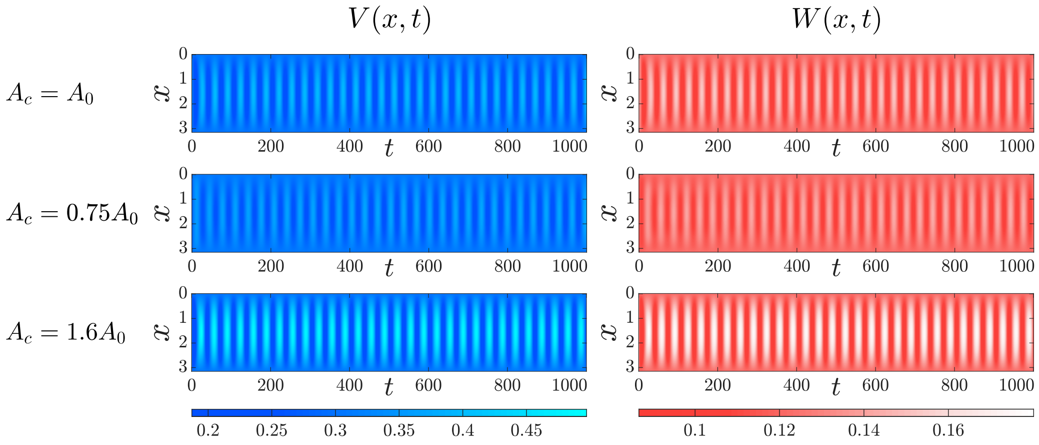

Figure 14: Independent amplitude coordinations for the periodic oscillation in the F-N system with D.B.C. The first row shows the original oscillation without a coordinator. In the second row, the amplitude is suppressed ($r_A = 0.75$) by the coordinator $(f_{11}, f_{12}) = (-0.378, 0.239)$. In the last row, the amplitude is magnified ($r_A = 1.6$) by the coordinator $(f_{11}, f_{12}) = (0.48, -1.4134)$.

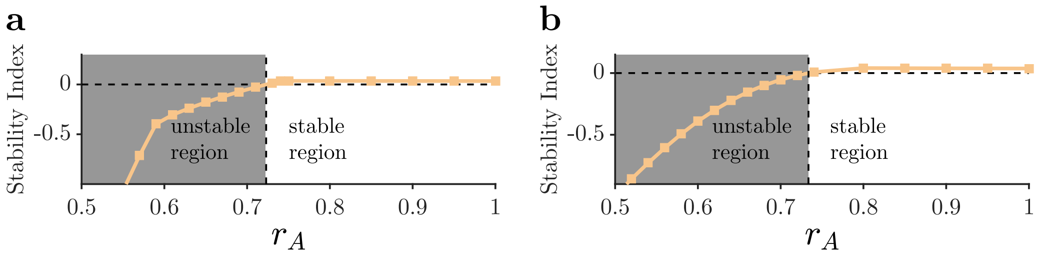

Figure 15: **a** Stability analysis for the amplitude coordination in the F-N system with N.B.C. **b** Stability analysis for the amplitude coordination in the F-N system with D.B.C. For every r_A, we computed the minimum of the corresponding stability index $c_0(k_i)$. If it is less than zero, then the coordinated periodic oscillation becomes unstable.

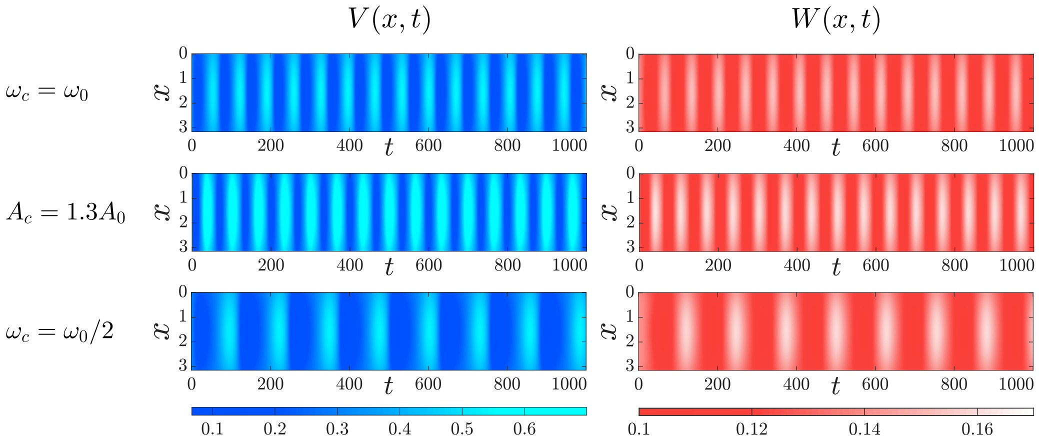

Figure 16: Independent amplitude and frequency coordinations for the “bigger” periodic oscillation (at $\epsilon = 0.01$) in the F-N system with R.B.C. The first row shows the original oscillation without a coordinator. In the second row, the amplitude is magnified ($r_A = 1.3$) by the coordinator $(f_{11}, f_{12}) = (0.24, -1.8739)$. In the last row, the frequency is halved by the coordinator $(f_{11}, f_{12}) = (-0.032, 0.4284)$.

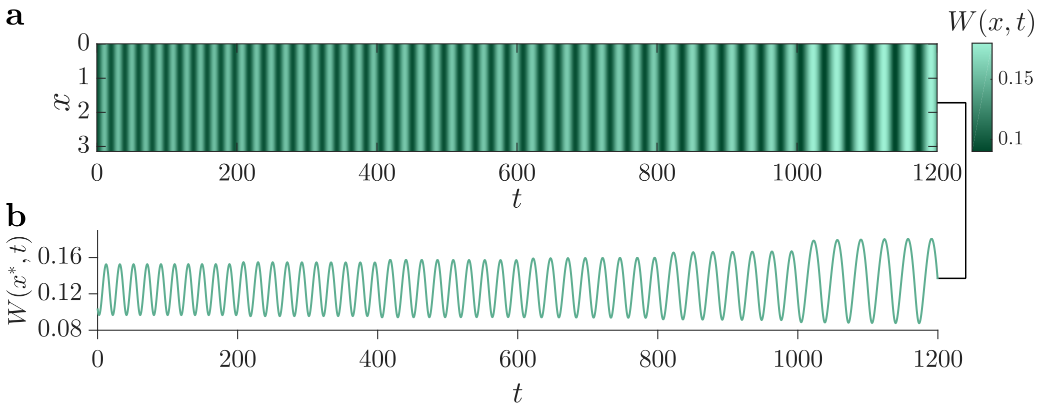

Figure 17: The time course of W for the hybrid coordination in the F-N system with N.B.C., which corresponds to the time course of V given in main text Fig. 7a, b. **b** The time course at $x^* = \pi/2$.

References

[1] Yi, F., Wei, J. & Shi, J. Bifurcation and spatiotemporal patterns in a homogeneous diffusive predator–prey system. *J. Differ. Equ.* **246**, 1944–1977 (2009).

[2] Kuznetsov, Y. *Elements of Applied Bifurcation Theory.* (Springer-Verlag, New York, 2004).

[3] Ge, T., Tian, X., Kurths, J., Feng, J. & Lin, W. Achieving modulated oscillations by feedback control. *Phys. Rev. E* **90**, 022909 (2014).

[4] Aguda, B. D., Kim, Y., Piper-Hunter, M. G., Friedman, A. & Marsh, C. B. MicroRNA regulation of a cancer network: consequences of the feedback loops involving miR-17-92, E2F, and Myc. *Proc. Natl Acad. Sci. USA* **105**, 19678–19683 (2008).

[5] Zheng Q., & Shen, J. Dynamics and pattern formation in a cancer network with diffusion. *Communications in Nonlinear Science and Numerical Simulation*, **27**, 93–109 (2015).

[6] Novák, B. & Tyson, J. J. Design principles of biochemical oscillators. *Nat. Rev. Mol. Cell Biol.* **9**, 981–991 (2008).
