# Supplementary material for: A frequency-amplitude coordinator and its optimal energy consumption for biological oscillators
Source: Nat Commun. 2021 Oct 8;12:5894. doi: 10.1038/s41467-021-26182-2 (PMC8501100; doi:10.1038/s41467-021-26182-2)
Supplement: Supplementary file 2 — Description of Additional Supplementary Files [file 41467_2021_26182_MOESM2_ESM.pdf]

Title: Supplementary Movie 1

Description: This animation shows the independent frequency coordination of the periodic oscillation in the F-N system with zero flux at the boundaries. The two coefficients for the applied coordinator are shown in the top two panels. In the bottom, we see that, as the coordination varies (the moving circle), the frequency is decreased while the amplitude is almost unchanged.

Title: Supplementary Movie 2

Description: This animation shows the independent frequency coordination of the periodic oscillation in the F-N system with fixed value at the boundaries. The two coefficients for the applied coordinator are shown in the top two panels. In the bottom, we see that, as the coordination varies (the moving circle), the frequency is decreased while the amplitude is almost unchanged.

Title: Supplementary Movie 3

Description: This animation shows the independent frequency coordination of the periodic oscillation in the F-N system with the Robin boundary condition. It can be regarded as the intermediate case between the case shown in Movie 1 and 2. For this case, the two coefficients for the coordinator are shown in the top two panels. In the bottom, we see that, as the coordination varies (the moving circle), the frequency is decreased while the amplitude is almost unchanged.

Title: Supplementary Movie 4

Description: This animation shows the independent amplitude coordination of the periodic oscillation in the F-N system with zero flux at the boundary (N.B.C.). As the coordinator varies (the moving circle), the amplitude is suppressed (the color becomes darker) while the frequency is almost unchanged. For convenience, we also show the time course at $x^* = \pi/2$ in the bottom.

Title: Supplementary Movie 5

Description: This animation shows the independent amplitude coordination of the periodic oscillation in the "cancer network". With different coordinating policies (the moving circle) shown in the top panels, the amplitude of the protein concentration is suppressed (bottom panel). Note that A_0/A_c equals the reciprocal of r_A.

Title: Supplementary Movie 6

Description: This animation shows the phase space evolution of the time course in Fig. 7b. Six panels show the evolution in different time intervals. Obviously, due to the hybrid coordination, the amplitude (size of the circle) and the frequency are different in these panels.
