# Supplementary figures and images for: A frequency-amplitude coordinator and its optimal energy consumption for biological oscillators
Source: Nat Commun. 2021 Oct 8;12:5894. doi: 10.1038/s41467-021-26182-2 (PMC8501100; doi:10.1038/s41467-021-26182-2)

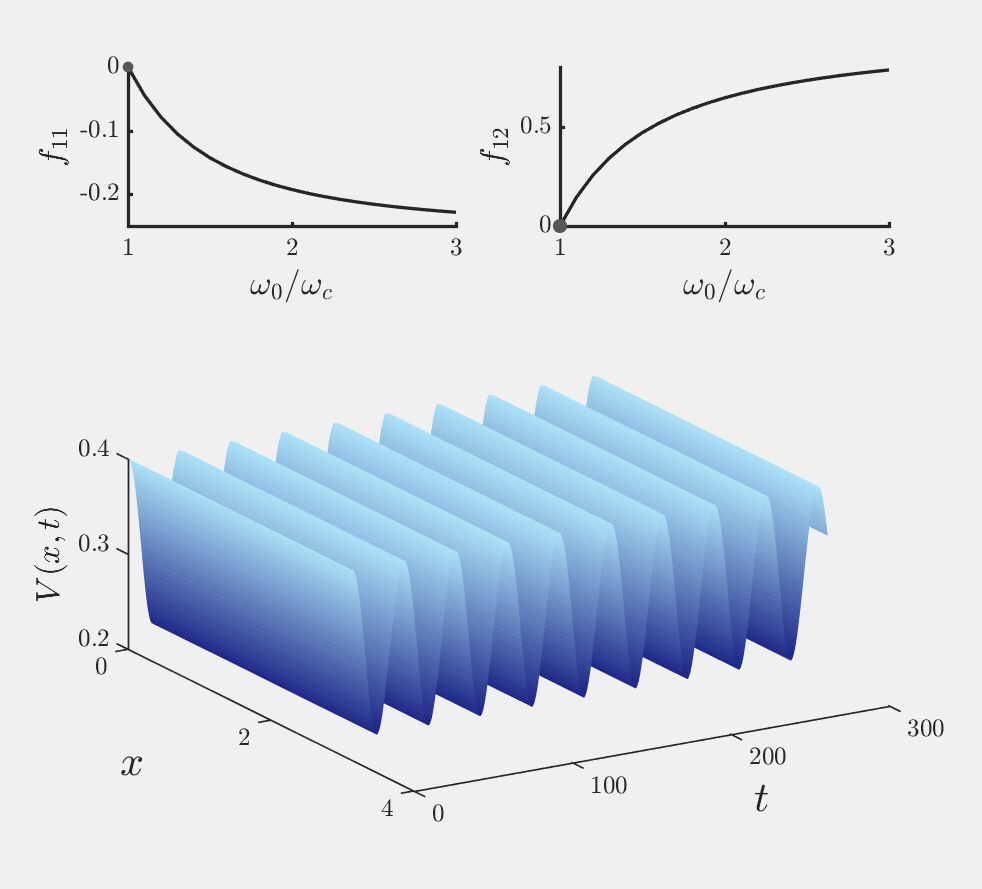

Supplement: Supplementary file 3 — Supplementary Movie 1 [file 41467_2021_26182_MOESM3_ESM.gif]

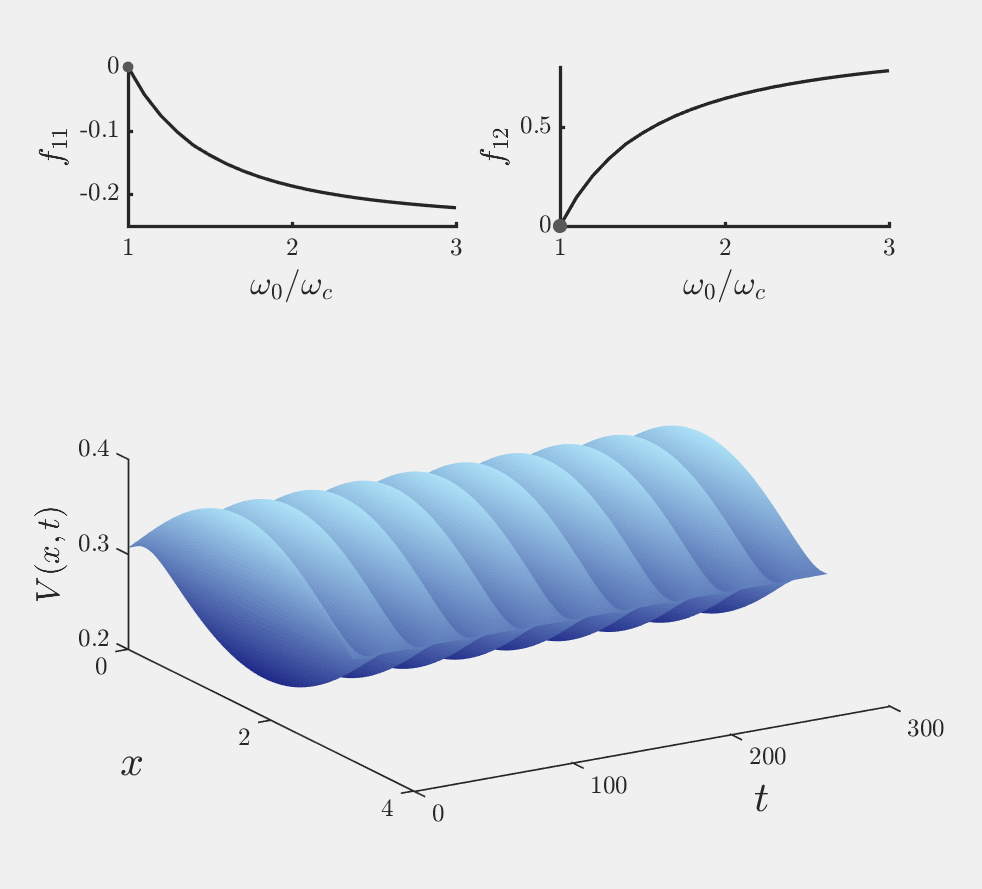

Supplement: Supplementary file 4 — Supplementary Movie 2 [file 41467_2021_26182_MOESM4_ESM.gif]

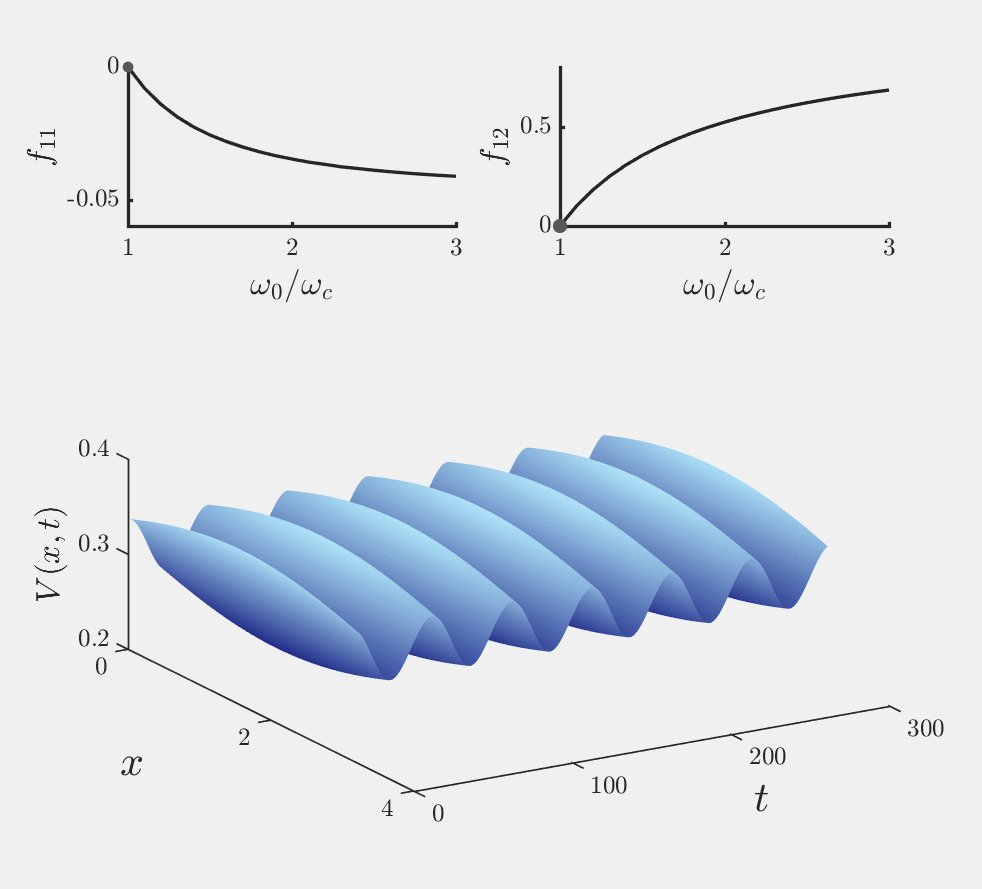

Supplement: Supplementary file 5 — Supplementary Movie 3 [file 41467_2021_26182_MOESM5_ESM.gif]

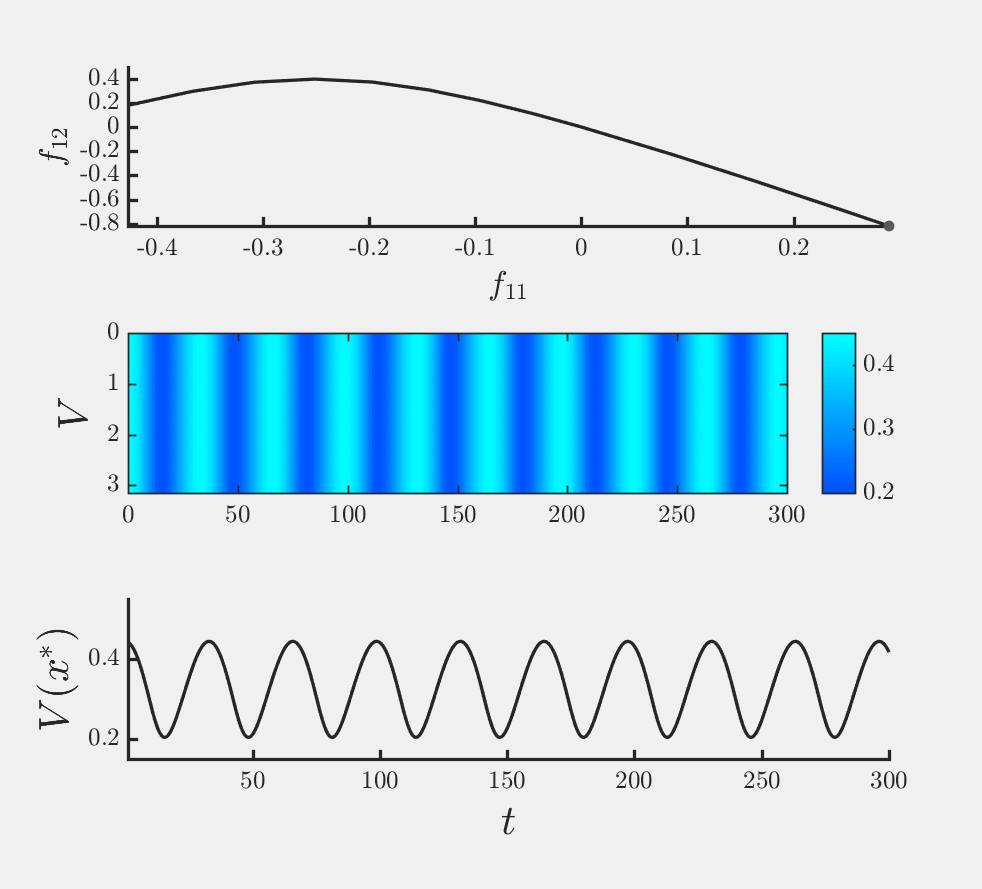

Supplement: Supplementary file 6 — Supplementary Movie 4 [file 41467_2021_26182_MOESM6_ESM.gif]

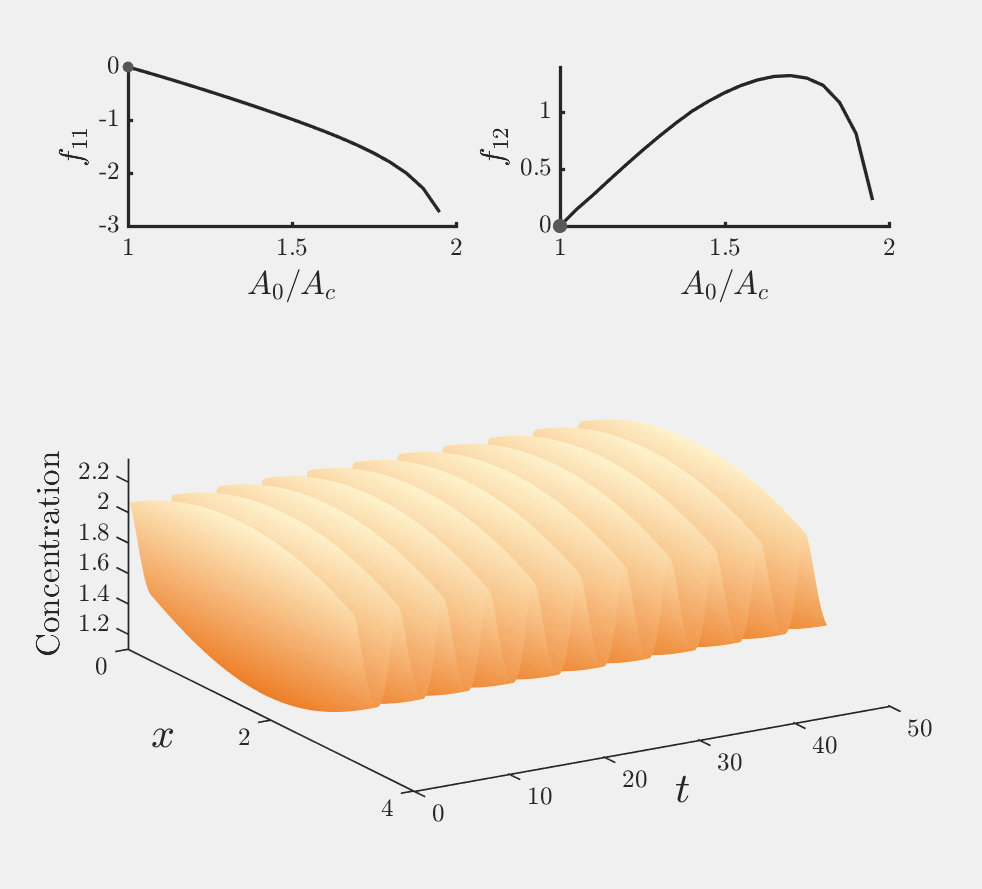

Supplement: Supplementary file 7 — Supplementary Movie 5 [file 41467_2021_26182_MOESM7_ESM.gif]

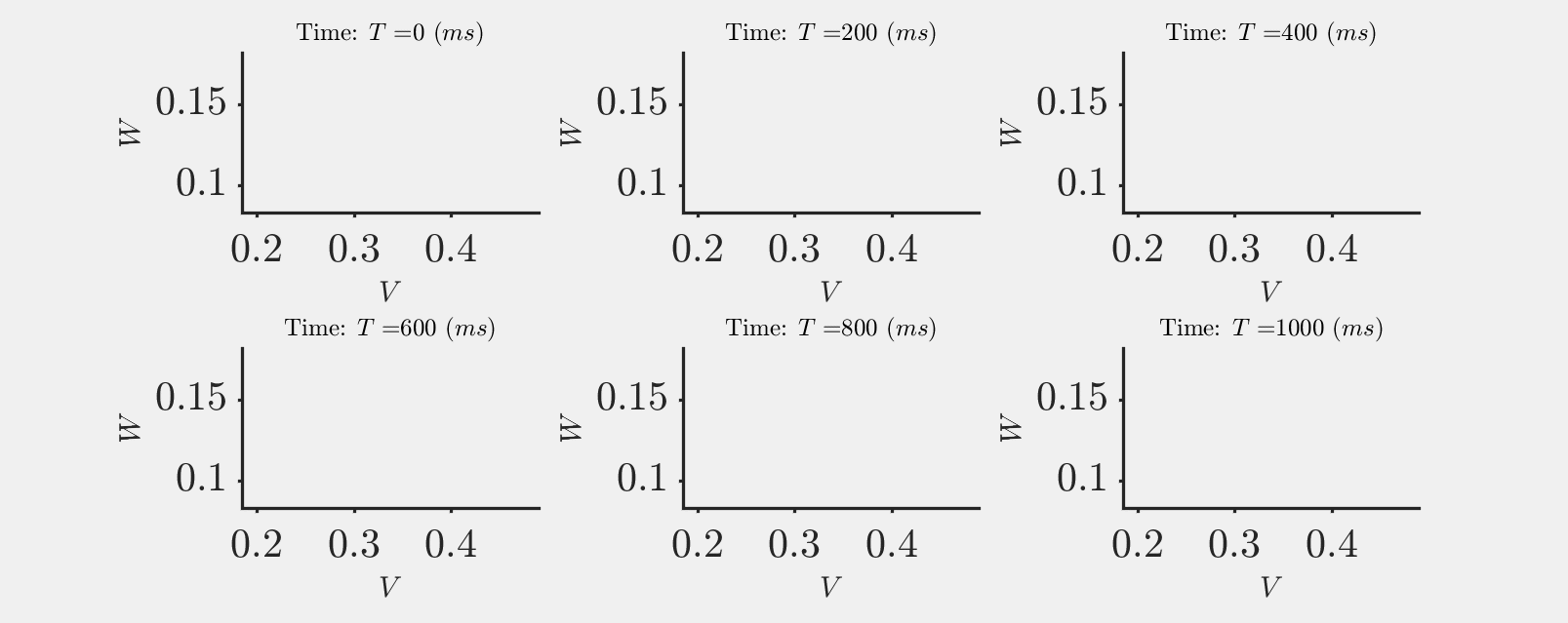

Supplement: Supplementary file 8 — Supplementary Movie 6 [file 41467_2021_26182_MOESM8_ESM.gif]
